# A Translational Paradigm to Study the Effects of Uncontrollable Stress in Humans

**DOI:** 10.3390/ijms21176010

**Published:** 2020-08-20

**Authors:** Laura E. Meine, Katja Schüler, Gal Richter-Levin, Vanessa Scholz, Michele Wessa

**Affiliations:** 1 Department of Clinical Psychology and Neuropsychology, Institute of Psychology, Johannes Gutenberg-University Mainz, 55122 Mainz, Germany; laura.meine@uni-mainz.de (L.E.M.); katja.schueler@kgu.de (K.S.); v.scholz@donders.ru.nl (V.S.); 2 Department of Psychology, University of Haifa, Haifa 3498838, Israel; glevin@univ.haifa.ac.il; 3 Sagol Department of Neurobiology, University of Haifa, Haifa 3498838, Israel; 4 The Integrated Brain and Behavior Research Center (IBBR), University of Haifa, Mount Carmel, Haifa 3498838, Israel; 5 Donders Institute for Brain, Cognition and Behaviour, Radboud University, 6525 HR Nijmegen, The Netherlands; 6 Leibniz Institute for Resilience Research, Research Group Wessa, 55122 Mainz, Germany

**Keywords:** uncontrollable stress, learned helplessness, control, translational research, resilience

## Abstract

Theories on the aetiology of depression in humans are intimately linked to animal research on stressor controllability effects. However, explicit translations of established animal designs are lacking. In two consecutive studies, we developed a translational paradigm to study stressor controllability effects in humans. In the first study, we compared three groups of participants, one exposed to escapable stress, one yoked inescapable stress group, and a control group not exposed to stress. Although group differences indicated successful stress induction, the manipulation failed to differentiate groups according to controllability. In the second study, we employed an improved paradigm and contrasted only an escapable stress group to a yoked inescapable stress group. The final design successfully induced differential effects on self-reported perceived control, exhaustion, helplessness, and behavioural indices of adaptation to stress. The latter were examined in a new escape behaviour test which was modelled after the classic shuttle box animal paradigm. Contrary to the learned helplessness literature, exposure to uncontrollable stress led to more activity and exploration; however, these behaviours were ultimately not adaptive. We discuss the results and possible applications in light of the findings on learning and agency beliefs, inter-individual differences, and interventions aimed at improving resilience to stress-induced mental dysfunction.

## 1. Introduction

“Don’t stress over what you can’t control.”—a common piece of advice that seems easier said than done. In fact, empirical evidence shows that lack of control over a situation makes it all the more stressful [1]. Human stress theories, as well as extensive animal research, have suggested and compellingly demonstrated how controllability over an aversive event significantly modulates the corresponding reaction and appraisal [2].

In human research, stressor controllability has thus far mostly been discussed in the context of depression pathogenesis, referring to the theory of learned helplessness [2,3,4]. The experience of uncontrollable stress induces a failure to escape aversive stimulation, alongside increased passivity and anxiety, overall termed learned helplessness (LH). In the 1960s, Seligman and Maier [4] developed the triadic design which compares three groups of subjects, one exposed to escapable electric shocks, another exposed to inescapable shocks, and a third group not receiving any aversive stimulation. Importantly, animals in the inescapable stress group are yoked to animals in the escapable condition, in order to ensure that both groups receive precisely the same number, duration, and overall pattern of shocks. Thus, the effects of controllability can be separated from mere stress effects. Subjects are subsequently tested in a novel environment, which exposes them to easily escapable shocks (shuttle box paradigm; [4,5,6]). Results consistently showed that, in contrast to the other groups, animals which had previously experienced inescapable stress, now failed to escape. Fifty years of research more thoroughly investigating this finding revealed that animals showed symptoms of greater passivity, reduced social interaction, neophobia, and decreased food intake in response to inescapable stress [2]—behaviours mirrored in the symptoms of anxiety and depression in humans [3]. Indeed, studies in humans linked the lack of control over a stressor with increased subjective stress [7], anxiety [8], pain intensity perception [9], deficits in selective attention [10], and impaired fear extinction learning [11].

It has been pointed out previously that “LH is a proven translational concept” [3] (p. 244). In fact, as early as the 1970s, researchers applied the triadic design to humans, subjecting groups of university students to either controllable noise, uncontrollable noise, or no noise [12,13]. Thornton and Jacobs [14] employed electric shocks to induce differential effects of stressor controllability. Importantly, these early translations stayed close to the animal triadic design, focusing on proper yoking and even incorporating a test akin to the shuttle box. In particular, researchers assessed participants’ success in terminating noise bursts by moving a lever from side to side [12,13,15]. Exploiting the possibility for more complex cognitive testing, modifications of the triadic design were introduced which required participants to solve more demanding cognitive tasks in order to terminate aversive stimulation [13,16]. With the advent of more powerful neuroimaging tools, studies also investigated the neural correlates of stressor controllability in humans, focusing more on group differences during stress exposure rather than on subsequent affective, cognitive, or behavioural assessments. Promising findings suggest modulations in event-related potentials [17,18], in activation in e.g., the ventromedial prefrontal cortex (vmPFC; [19]) and the amygdala [20], as well as in connectivity measures [9]. Human researchers have veered in different directions to study particular aspects potentially implicated in or altered by stressor controllability. However, there still remains a gap in translational experimental study designs that incorporate the triadic stress induction design as well as measures assessing stressor controllability effects on affect, cognition, and behaviour. Whereas animal research can boast established, well-standardised paradigms [5], human studies are largely heterogeneous concerning their experimental setup and the outcome variables they investigate. To illustrate this, stressors ranged from physical stimuli, i.e., electric shocks [8,9,11], thermal stimulation [20], and loud sounds [7,10] to video clips of snakes [19] and unsolvable reasoning tasks [21]. Probably for reasons of feasibility, many studies opted for a within-subject design, rather than comparing three groups according to the triadic design. Salomons and colleagues [20] omitted a control group which did not receive any aversive stimulation, contrasting instead only two groups. Diener and colleagues [17] employed a forewarned design, i.e., participants in the controllable stress group were able to completely avoid stressful stimulation which led to imperfect yoking between conditions.

This lack of standardised procedures hampers ongoing efforts to fully characterise the mechanistic underpinnings of how stressor controllability modulates our response to stress and its consequences on behaviour, affect, and cognition. Because individuals’ affect and cognitive functioning can be more readily assessed in humans than in animals, human research has mainly focused on self-report and cognitive tasks to assess effects of stressor controllability. In contrast, animal studies mostly rely on behavioural read-outs. Therefore, broader analyses of stressor controllability effects in humans appear useful in strengthening translational approaches.

To this end, we sought to fill the gap in translational studies using a paradigm which more closely translates the established triadic animal design and which provides a feasible means to investigate stressor controllability effects in humans on an affective, cognitive, and behavioural level. In two consecutive studies, we developed, validated, and adapted an experimental paradigm to study stressor controllability effects in humans. In the first study, we compared three experimental groups: (1) an escapable stress group exposed to controllable stress (EC), (2) a yoked group exposed to uncontrollable stress (YC), and (3) a control group without stress exposure (CC). Our main hypotheses were related to effects of stressor controllability (and not stress, per se), however, we included a control group to validate our stress-induction procedure with respect to both the mere stress effects as well as effects of stressor controllability (manipulation check). In order to characterise these effects on multiple response levels, we assessed reaction times (RT) and subjective ratings on helplessness, exhaustion, and frustration during stress exposure, as well as post stressor changes in affect, escape behaviour, and working memory. With respect to the manipulation check, we verified that EC and YC rated the stressor as aversive and that perceived control ratings differentiated the three groups, with CC reporting highest perceived control, followed by EC, and YC rating lowest perceived control.

Many studies have reported detrimental effects of acute stress on e.g., affect [22,23] and cognition [24,25] in humans. Therefore, we expected both stress-exposed groups (EC and YC) to differ from CC in indices assessed during the stress exposure phase (subjective ratings, RT) as well as in subsequently measured affect, escape behaviour, and working memory. Our hypotheses were as follows: First, with respect to assessments during stress exposure, we expected YC to report highest exhaustion, frustration, and helplessness and show longest RT, followed by EC, then CC. Second, related to subsequent (post stress) measurements, we expected YC to show a greater increase in depressive symptoms, anxiety, and negative mood as well as a greater decrease in positive mood compared to EC. As CC was not exposed to stress, we expected no change in affective state in this group. For the escape behaviour test (comprising a stress-free exploration phase and subsequent exposure to a familiar stressor with the possibility to escape), we expected YC to show less exploration behaviour, fewer successful escapes from stress, and less efficiency in escaping stress compared to EC. Based on the animal literature, we expected CC to fall in between YC and EC with respect to successful escapes and escaping efficiency. Furthermore, we hypothesised that YC would perform worst in the working memory test, followed by EC, with CC scoring highest.

Although group differences indicated successful stress induction in the first study, the manipulation failed to differentiate groups according to controllability-related effects on subsequent measurements of affect, cognition, and escape behaviour. In the second study, we therefore employed an improved paradigm, comparing EC and YC only, and testing the same hypotheses described above. In particular, we examined subjective ratings of helplessness and exhaustion (omitting frustration due to time constraints), RT, changes in affect, and escape behaviour. Due to time constraints, working memory was not assessed in this study. Please refer to Section 4 for details on methodology.

## 2. Results

### 2.1. Study 1: Results of the Initial Paradigm

#### 2.1.1. A Priori Group Comparisons and Manipulation Check

**Figure 1:**
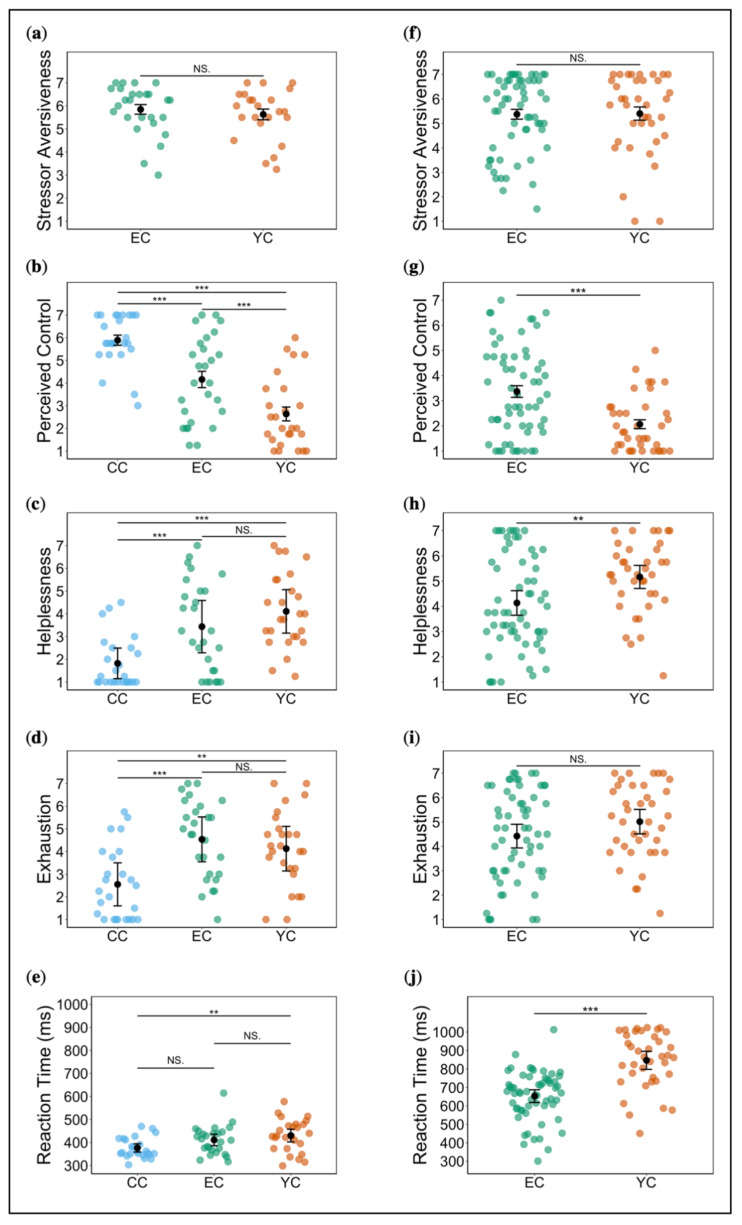
Ratings and reaction times during stress induction in study 1 (left panels) and study 2 (right panels). (**a**)/(**f**) Stressor aversiveness; (**b**)/(**g**) perceived control; (**c**)/(**h**) helplessness; (**d**)/(**i**) exhaustion; (**e**)/(**j**) reaction times. Error bars denote standard error (* *p* < 0.05, ** *p* < 0.01, *** *p* < 0.001).

We observed no a priori group differences with respect to sex, working memory IQ, and alertness. However, groups differed significantly in age (*X*^2^ = 6.52, *df* = 2, *p* < 0.05, *η*^2^*_H_* = 0.06) (Table 1). We identified two outliers which we excluded from subsequent analyses. Because of missing data, we compared stressor aversiveness ratings between 26 EC participants and 23 YC participants only. Both groups equally rated the stressor as very aversive (global *M* = 5.74; *F*(1, 47) = 0.48, *p* = 0.49, partial *η*^2^ = 0.01), suggesting successful stress induction (Figure 1a). We observed no significant habituation or sensitization effects, rather aversiveness ratings remained near-constant over trials (*F*(3, 141) = 0.97, *p* = 0.38, partial *η*^2^ = 0.02 (Greenhouse–Geisser corrected, *ε* = 0.64)). Perceived control varied significantly between all three groups (*F*(2, 74) = 29.67, *p* < 0.001, partial *η*^2^ = 0.45; all post hoc comparisons: *p* < 0.001) with CC reporting highest control, followed by EC, and YC reporting lowest control (Figure 1b). There was an effect of time (*F*(3, 222) = 3.47, *p* < 0.05, partial *η*^2^ = 0.04 (Greenhouse–Geisser corrected, *ε* = 0.63)), reflecting a slight decrease in perceived control across trials. See the Supplementary Materials for analysis of sex differences (Supplement 6).

#### 2.1.2. Ratings and RT during Acute Stress Exposure

Analysis of helplessness ratings indicated a significant effect of group (*X*^2^ = 26.18, *df* = 2, *p* < 0.001, *η*^2^*_H_* = 0.33), but post hoc comparisons showed significant differences only between stress and control, with both EC and YC reporting greater helplessness compared to CC (EC vs. CC and YC vs. CC: *p* < 0.001). A comparison of EC and YC did not reach significance (*p* = 0.12), but we noted differences in the expected direction (Figure 1c). Similarly, analysis of mean exhaustion ratings supplied during stress exposure revealed a significant effect of group (*F*(2, 75) = 10.24, *p* < 0.001, partial *η*^2^ = 0.21). However, post hoc comparisons showed significant differences only between stress and control with higher ratings in both EC and YC compared to CC (EC vs. CC: *p* < 0.001 and YC vs. CC: *p* < 0.01). EC and YC did not differ (*p* = 0.37) (Figure 1d). Regarding frustration, we also noted significant group differences (*X*^2^ = 16.75, *df* = 2, *p* < 0.001, *η*^2^*_H_* = 0.20), but post hoc comparisons showed that only YC and CC differed significantly (*p* < 0.001), whereas EC vs. CC and EC vs. YC comparisons revealed only a trend effect (both *p* = 0.07). As anticipated, YC reported greatest frustration, followed by EC, and then, CC (see Supplement 1).

One CC participant who did not react to any cue and one EC outlier were excluded from RT analysis. We observed an effect of group (*F*(2, 73) = 4.98, *p* < 0.01, partial *η*^2^ = 0.12). As expected, CC was fastest, followed by EC, with YC being slowest (Figure 1e). However, post hoc comparisons were not significant for EC vs. CC (*p* = 0.10) and EC vs. YC (*p* = 0.26). The difference between YC and CC was significant at *p* < 0.01. See the Supplementary Materials for analysis of changes across trials and sex differences (Supplement 7).

#### 2.1.3. Affective State, Escape Behaviour, and Cognitive Performance during Subsequent (Post Stress) Assessment Changes in Affective State

**Figure 2:**
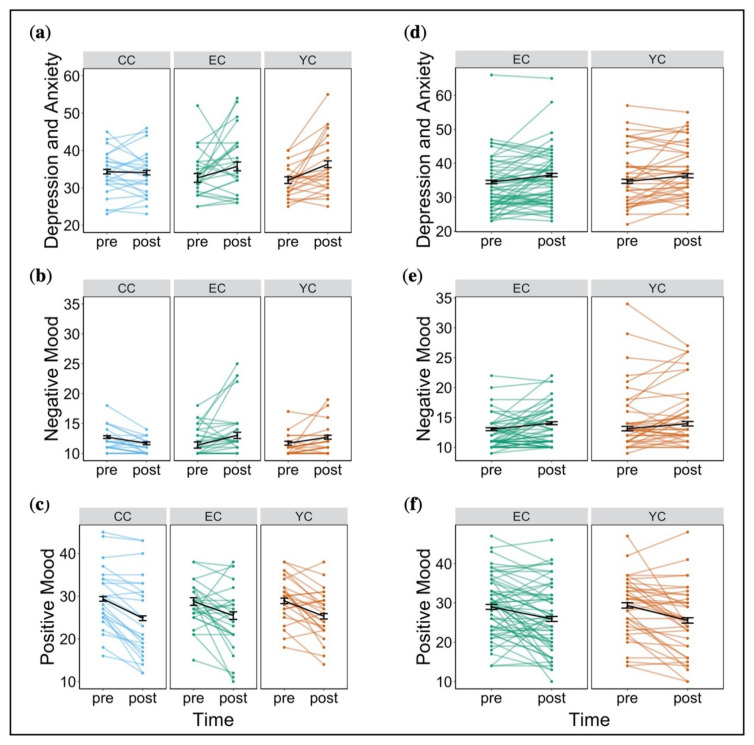
Changes in affective state from pre to post stress induction in study 1 and study 2. (**a**) Depressive symptoms and anxiety in study 1. (**b**) Negative mood in study 1. (**c**) Positive mood in study 1. (**d**) Depressive symptoms and anxiety in study 2. (**e**) Negative mood in study 2. (**f**) Positive mood in study 2. Error bars denote standard error (* *p* < 0.05, ** *p* < 0.01, *** *p* < 0.001).

Two participants per group were missing data on depressive state and anxiety and had to be excluded from this analysis. Additionally, we removed one outlier who reported extremely high depressive state and anxiety before stress exposure. There was no significant effect of group (*F*(2, 68) = 0.49, *p* = 0.62, partial *η*^2^ = 0.01) on state of depression and anxiety, but we observed a significant effect of time (*F*(1, 68) = 9.38, *p* < 0.01, partial *η*^2^ = 0.12). This reflected an increase in depressive symptoms and anxiety from pre to post stress induction. Following correction for family-wise error, there was no significant interaction of group x time (*F*(2, 68) = 3.02, *p* = 0.39, partial *η*^2^ = 0.08) on depressive symptoms and anxiety (Figure 2a). With respect to negative mood, ratings from four participants were missing and were thus, excluded from analysis. We observed a significant interaction of group x time on negative mood (*F*(2, 68) = 6.27, *p* < 0.05, partial *η*^2^ = 0.16), reflecting an increase in negative mood across stress groups (EC and YC), albeit only significant in EC. CC, rather, showed a decrease in negative mood, though this was not significant (Figure 2b). With regard to positive mood, there was no significant effect of group (*F*(2, 75) = 0.29, *p* = 0.75, partial *η*^2^ = 0.008), but a significant effect of time (*F*(1, 75) = 42.39, *p* < 0.001, partial *η*^2^ = 0.36). Positive mood decreased from pre to post stress induction across groups (Figure 2c). No interaction of group x time emerged (*F*(2, 75) = 0.39, *p* = 0.68, partial *η*^2^ = 0.01). For analysis of sex differences see Supplement 8.

##### Escape Behaviour

We investigated exploration during the stress-free phase, i.e., the number of unique spaces visited per minute. Results revealed no group differences (*X*^2^ = 2.60, *df* = 2, *p* = 0.27, *η*^2^*_H_* = 0.002) (see Supplement 2). Next, we compared the total sum of successful escapes during the stress phase between groups and found no significant differences (*X*^2^ = 0.95, *df* = 2, *p* = 0.62, *η*^2^*_H_* = −0.01) (see Supplement 3). Finally, we examined behavioural efficiency as the quotient of moves per minute and sum of escapes across trials. Again, no significant group differences emerged (*X*^2^ = 1.54, *df* = 2, *p* = 0.46, *η*^2^*_H_* = −0.006) (see Supplement 4). The striking lack of group differences in escape behaviour was contrary to our hypotheses. For analysis of sex differences see Supplement 8.

##### Working Memory

We analysed working memory in terms of partial-credit load in the reading span task. Four participants had missing data and had to be excluded from analysis. We observed significant group differences in partial-credit load scores (*F*(2, 71) = 6.67, *p* < 0.05, partial *η*^2^ = 0.16). Contrary to our hypothesis, EC performed significantly worse than CC (*p* < 0.01). The differences between EC and YC (*p* = 0.11) as well as CC and YC (*p* = 0.11) were not significant (see Supplement 5). For analysis of sex differences see Supplement 8.

### 2.2. Study 2: Results of the Improved Paradigm

#### 2.2.1. A Priori Group Comparisons and Manipulation Check

We observed no a priori group differences in sex, age, or level of education (Table 2). One participant had missing stressor aversiveness data and was excluded from this analysis. EC and YC both rated the stressor as very aversive (global *M* = 5.38; *F*(1, 95) = 0.007, *p* = 0.93, partial *η*^2^ = 0.0004; Figure 1f). There was a trend effect of time, reflecting a slight increase in reported aversiveness over trials (*F*(3, 285) = 2.59, *p* = 0.07, partial *η*^2^ = 0.03 (Greenhouse–Geisser corrected, *ε* = 0.74)). Groups differed significantly with respect to perceived control (*F*(1, 96) = 16.23, *p* < 0.001, partial *η*^2^ = 0.14), with YC showing lower ratings, as hypothesised (Figure 1g). There was a significant effect of time on controllability ratings, indicating a decrease over trials (*F*(3, 288) = 6.70, *p* < 0.001, partial *η*^2^ = 0.07 (Greenhouse–Geisser corrected, *ε* = 0.84)). Contrary to study 1, EC participants were required to figure out for themselves how to exert control and terminate the aversive stimulation. We examined rates of correct performance to assess whether this was in line with our intended manipulation. Aside from eight participants with rates below 30%, EC participants performed tolerably well (*M* = 0.60, *SD* = 0.49). We ascertained that participants with low performance did not substantially affect subsequent analyses before we decided to include them. For analysis of sex differences see Supplement 9.

#### 2.2.2. Ratings and RT during Acute Stress Exposure

YC reported significantly greater feelings of helplessness compared to EC (*t*(96) = −2.90, *p* < 0.01, −*d* = 0.60; Figure 1h). Analysis of exhaustion ratings revealed no significant group difference (*t*(96) = −1.62, *p* = 0.11, *d* = −0.34; Figure 1i). EC reacted significantly faster than YC and a large effect was indicated (*t*(96) = −6.67, *p* < 0.001, *d* = −1.38; Figure 1j). See the Supplementary Materials for analysis of changes across trials and sex differences (Supplement 10).

#### 2.2.3. Affective State and Escape Behaviour during Subsequent (Post Stress) Assessment

##### Changes in Affective State

We observed a trend for significant group differences (*F*(1, 96) = 3.09, *p* = 0.08, partial *η*^2^ = 0.03) with respect to combined depressive state and anxiety. YC displayed overall higher scores compared to EC. There was an increase in scores from pre to post stress exposure across groups (*F*(1, 96) = 11.13, *p* < 0.01, partial *η*^2^ = 0.10) but no interaction of group x time (*F*(1, 96) = 0.14, *p* = 0.71, partial *η*^2^ = 0.001) (Figure 2d). With regard to negative mood, we observed a significant difference between groups (*F*(1, 96) = 7.10, *p* < 0.01, partial *η*^2^ = 0.07). YC showed higher negative mood scores both at pre and post stress exposure. Both groups reported an increase in negative mood from pre to post stress exposure (*F*(1, 96) = 8.83, *p* < 0.01, partial *η*^2^ = 0.08). We found no interaction of group x time on negative mood (*F*(1, 96) = 0.08, *p* = 0.78, partial *η*^2^ = 0.0008) (Figure 2e). EC and YC did not differ in relation to positive mood (*F*(1, 96) = 0.08, *p* = 0.77, partial *η*^2^ = 0.003) but there was a significant effect of time (*F*(1, 96) = 25.72, *p* < 0.001, partial *η*^2^ = 0.21), indicating a decrease in positive mood from pre to post stress exposure in both groups. Again, there was no interaction between group x time (*F*(1, 96) = 0.30, *p* = 0.58, partial *η*^2^ = 0.003) (Figure 2f). Thus, we observed no evidence of differential change in affective state related to controllability. For analysis of sex differences see Supplement 11.

##### Escape Behaviour

**Figure 3:**
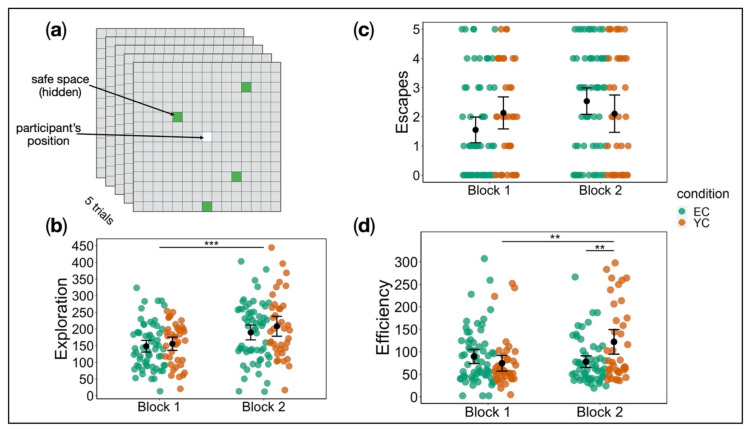
(**a**) Escape behaviour test (one block). (**b**) Exploration during the stress-free phase in the escape behaviour test by group and block. Both groups explored more in block 2 compared to block 1; we observed no interaction effects. (**c**) Escapes from stress in the escape behaviour test by group and block. EC escaped the stressor more often in block 2 compared to block 1, whereas no such improvement was evident in YC. Despite that, the interaction of group x block did not survive family-wise error correction. (**d**) Interaction effect of group x block on efficiency in the escape behaviour test. Whereas EC and YC did not differ in efficiency in block 1, YC showed significantly less efficient behaviour (higher scores) in block 2 compared to EC. Error bars denote standard error. Only significant effects are indicated (* *p* < 0.05, ** *p* < 0.01, *** *p* < 0.001).

We analysed stress-free exploration behaviour and observed no significant effect of group (*F*(1, 96) = 0.74, *p* = 0.39, partial *η*^2^ = 0.008). A significant effect of block indicated an increase in exploration from block 1 to block 2 across groups (*F*(1, 96) = 48.58, *p* < 0.001, partial *η*^2^ = 0.34). There was no interaction of group x block (*F*(1, 96) = 0.70, *p* = 0.40, partial *η*^2^ = 0.007) (Figure 3b). Analysis of escape rates revealed an interaction of group x block, but this effect did not survive correction for family-wise error (*F*(1, 96) = 5.11, *p* = 0.16, partial *η*^2^ = 0.05) (Figure 3c). Finally, investigation of escaping efficiency, i.e., the trade-off between level of activity and escape rate, showed a significant interaction of group x block (*F*(1, 91) = 12.38, *p* < 0.001, partial *η*^2^ = 0.12). Whereas EC and YC did not differ in efficiency in block 1 (*p* = 0.51), YC showed significantly less efficient behaviour in block 2 compared to EC (*p* < 0.01). While the behaviour of YC participants represented a considerable performance decrement (block 1 vs. block 2: *p* < 0.01), participants in EC even improved slightly from block 1 to block 2, albeit not statistically significant (*p* = 0.51) (Figure 3d). For analysis of sex differences see Supplement 11.

## 3. Discussion

In two consecutive studies, we developed and validated a translational paradigm to induce controllable and uncontrollable stress in humans and examined the effects of this manipulation on affective state, escape behaviour, and working memory. We hypothesised that exposure to uncontrollable stress would lead to longer RT; higher ratings of helplessness, exhaustion, and frustration; greater increase in negative affect; less efficient escape behaviour; and poorer working memory performance compared to controllable stress. In study 1, we included a control group which was not exposed to any aversive stimulation and served to establish the stressfulness of our stressor.

In study 1, participants in both stress groups (EC and YC) perceived equally the stressor as very aversive and showed no habituation over trials. As expected, our results demonstrated a robust stress effect, evidenced by higher levels of exhaustion, frustration, greater increases in depressive symptoms, anxiety, and negative mood in both EC and YC compared to CC. However, stress effects were not evident with regard to working memory performance and escape behaviour. EC performed worst in the working memory task, but we had hypothesised YC would show the lowest scores. As working memory has previously been found to be impaired by acute stress [24] and lack of control [26], this contradictory result may reflect problems with our manipulation of stressor controllability. Perhaps, EC experienced greater strain due to higher task demands compared to YC during the stress induction. Correspondingly, EC reported feeling slightly more exhausted than YC, albeit not statistically significant. Indeed, analysis of group differences only partly supported our hypotheses with respect to stressor controllability effects. Even though a manipulation check indicated differential effects on perceived control as hypothesised, stress effects were evidently not exacerbated by lack of control. Observed group differences across read-outs were largely limited to stress groups vs. control, while direct comparisons between EC and YC yielded few significant results. This suggests that the manipulation of stressor controllability was not successful. It is of note though that longer RT and greater frustration and helplessness in YC compared to EC were in line with our hypotheses. The paradigm used in study 1, thus, provided a promising basis upon which to improve in study 2.

Two features of the stress induction procedure specifically seemed to be likely candidates for modification. First, our design required EC participants to endure a certain amount of aversive stimulation, until the cue signalled the option to terminate the stressor. Thus, they experienced similar lack of control to YC, at least in the beginning of trials. Other studies have circumvented this issue by employing a forewarned design which allows participants to completely avoid aversive stimulation [17]. However, as we were specifically interested in studying how control experience may moderate stress effects, reducing stress exposure in EC was not an option. Stress constitutes a pervasive fact of life [27,28] from which it is hard to escape. This rings true, especially in today’s society, where we are increasingly expected to multitask, show proficiency in a variety of subjects, and to shoulder ever more responsibility. Therefore, the study of factors or mechanisms which may moderate stress effects presents a more ecologically valid avenue of research than promoting total stress avoidance. Moreover, this feature of the paradigm represents an important parallel to commonly used animal designs and serves the purpose of translational research [5].

Second, given that EC participants could easily achieve perfect performance on the button-press task, they hardly ever experienced aversive stimulation up to the maximum duration of 15 s. Therefore, they lacked a negative reference to compare to successfully shortened stress durations in trials in which they responded fast enough. A large body of research on learning and decision-making emphasises the importance of action–outcome contingencies [29,30]. By reacting to novel stimuli and observing the consequences, individuals can infer the level of control they have over the respective outcome. An outcome that is contingent on an action results in a high-agency estimate, whereas a non-contingent outcome is suggestive of low controllability and thus, fosters a belief in low agency [29]. It becomes clear, therefore, that the experience of both reward and punishment (or a lack of reward) is critical [31]. In study 2, we aimed at facilitating the observation of contingency of the outcome (stress stops vs. stress continues) on the action (button press vs. miss), thereby enhancing perceived control in EC. We expected that such a modification would suffice to render the manipulation of stressor controllability more successful, i.e., lead to significant differences in read-outs between EC and YC. Note that stressfulness of the aversive stimulation was established in study 1; hence, study 2 did not include a control group and the investigation of stressor aversiveness ratings was, thus, perfunctory only.

Results of study 2 on stressor aversiveness ratings were in line with study 1. As hypothesised, YC participants reported feeling less in control than EC participants. Not surprisingly, this EC group reported slightly lower perceived control compared to EC in study 1. Contrary to study 1, EC participants in study 2 were required to figure out through trial and error which of three possible buttons to press to terminate the stressor. They were, thus, bound to initially give some wrong responses and experience continued stress exposure which may have dampened perceived control. On the whole, however, the group difference in perceived control reflected a considerably larger effect compared to study 1. This suggests that enhancing action–outcome contingencies crucially improved our manipulation of stressor controllability. Robust group differences in the expected direction were now evident, with YC showing longer RT and higher ratings of exhaustion and helplessness. These findings point to crucial improvements in the manipulation of stressor controllability. In line with study 1, however, stressor controllability did not differentially affect changes in affective state. Results on pre to post stress induction depressive state, anxiety, and positive and negative mood highlight the overall negative effect that stress exerts on an individual’s affective state. While the two groups were not perfectly comparable in their negative mood scores before stress induction, it seems that stress here generally overrode any putative differences with respect to perceived control. Taken together, the findings from study 1 and 2 suggest that self-reported affective state—while certainly a valid measure with respect to stress reactivity—may not be the most sensitive indicator of differential control experience. It would have been interesting to have also investigated effects on working memory, but no such measurement was included in study 2. Substantial group differences in the escape behaviour test further corroborate the notion that objective, performance-based indices are better discriminated. YC showed behavioural patterns markedly different from EC, as both, again, tried to escape aversive stimulation in the grid-navigation task. However, EC and YC showed differences specifically in efficiency rather than in the more conventional measurements of exploration and escape rate. Although we did observe a tendency for differential escaping, the analysis of efficiency as the trade-off between level of activity and escape rate proved to be more sensitive. Differences between groups are clearly linked to the introduction of an element of lack of control represented by the unannounced relocation of safe spaces following the first five trials (block 1). Whereas EC seemed to recover quite quickly and even improved their escape rate from block 1 to block 2, YC showed considerable performance decrements. Based on a large body of LH research [2], we had anticipated that YC participants would show rather passive, resigned behaviour in response to renewed stress exposure. Despite their efforts, YC participants were not very successful in escaping the stressor. They ineffectively squandered resources for strategic thought on frantic searches of the grid. In fact, studies investigating learning and decision-making have demonstrated how stress renders individuals less able to critically evaluate potential actions and consequences in order to best achieve a present goal [25,32,33,34]. Similarly, studies have linked impulsive behaviour to stress history and psychopathology [35,36].

Individual differences have long been the focus of research on stress and control. Schönfeld and colleagues [37] recently discussed dimensional effects of self-efficacy on stress response, noting that high subjective control beliefs are not always beneficial. Bollini and colleagues [7] showed that locus of control moderated the association between perceived control over a stressor and related cortisol output. Generally, we did not have a sufficient sample size to investigate variation in the individual stress response or to explore moderating factors, nor was this our focus. We would be interested in examining individual differences in future studies. In study 2, we have included a larger number of EC participants to address the finding that not all EC participants were successful in escaping the stressor, even though it was objectively possible. However, the assessment of a sufficient number of participants (*N* > 80) was not possible within the duration of the project. Psychopathology research increasingly focuses on individual differences [38]. For instance, most individuals do not develop post-traumatic stress disorder following a single exposure to trauma. In fact, a traumatic event alone is seldom sufficient to induce clinical symptoms; more often, it is a combination of a traumatic event and additional risk factors [39]. To address individual differences in response to traumatic stress, recent research in rodents suggests incorporating individual predispositions and behavioural profiling [39].

Future studies could use our paradigm to investigate clinical populations, seeing as LH is widely considered a key concept in the aetiology of depression. Nonetheless, research on the mechanisms underlying LH is largely limited to the animal domain. Uncontrollable stress affects individuals across a variety of species [3] and has detrimental effects on a broad spectrum of functions [1]. Yet, differences in the experimental design of animal and human studies remain a challenge for translational research [40]. In this study, we successfully developed a translational paradigm to investigate effects of uncontrollable stress in humans. In keeping with early translational studies [13,14,15], the design unifies several essential features of the animal triadic design, i.e., use of a physical stressor, yoking of stress exposure, the operationalisation of control in terms of stressor termination, not prevention, a relatively simple task by which to exert control, and the assessment of escape behaviour [5]. However, our paradigm critically expands read-outs to encompass affective state, cognitive performance, and behaviour. Although we did not observe robust group differences in changes in affective state and did not assess cognitive performance in study 2, our paradigm allows for flexible testing of a range of read-outs. Naturally, stress effects in animal research are commonly assessed through behavioural indices after stress induction. Consequently, early translational studies focused on examining behavioural read-outs [13,14,15], emulating the rather simple tasks employed in animal research [4,5,6]. Making use of modern methods, recent research has largely concentrated on cognitive read-outs [10,11,25]. Our escape behaviour test translates indices traditionally used in animal studies (e.g., exploration and escape rate) to humans. However, considering that a certain level of complexity strengthens compliance and induces interesting variance in the data, our task markedly differs from early setups. Translational research often involves finding the sweet spot between rigorously imitating the original animal paradigm and allowing for obvious differences between species. With this in mind, the design of our escape behaviour test permits back-translation into animals. It, thus, contributes to bridging the gap in translational research between animal models and clinical practice [41,42].

This gap is particularly evident in research on stressor controllability. Animal researchers have, for a long while, shifted focus from investigation of LH to studying the experience of control. In a series of experiments [2,43,44,45], they could show compellingly that experience of control over a stressor protects against the negative consequences of later uncontrollable stress. Neurobiologically, this stress immunisation is thought to reflect persistent changes in pathways connecting the vmPFC with the dorsal raphe nucleus [2]. With increasing mechanistic insight, researchers have already started to develop pharmacological interventions to enhance perceived control [46,47]. However, it remains unclear how these findings translate to humans, as only a few studies have examined the neural underpinnings. Wanke and Schwabe [26] examined the effects of stressor controllability on working memory performance using an fMRI design to elucidate differential effects on a neural level. They report that perceived control, in contrast to objective control, altered prefrontal activation during the memory task. Unpublished work from our own group [48] showed increased vmPFC activation under controllable stress compared to uncontrollable stress—a finding that ties in with animal research.

In general, studies now increasingly focus on investigating putative protective factors and mechanisms which keep individuals mentally healthy, despite considerable stress exposure [27]. Our paradigm is well-suited to investigate both LH and stress immunisation. Observed group differences—especially in the escape behaviour test—lend support to control experience as a potent resilience mechanism.

There are, however, some limitations to consider. First, our results are based on a sample of healthy, rather well-educated, and relatively young adults. This was a necessary constraint to establish the paradigm. Future studies should investigate stressor controllability effects in more diverse samples and populations of increased vulnerability to LH, e.g., depressed patients. Second, while we verified that there was no difference in alertness as a baseline assessment of RT in study 1, no such a priori group comparison was conducted in study 2. Therefore, it is not clear whether participants in EC and YC showed inherently different baseline RT. Nevertheless, given the high degree of homogeneity of the sample, in terms of demographic variables, this should not pose a major limitation to interpreting the results. Third, we did not examine physiological or endocrine indicators of stress reactivity. Such measurements could offer additional mechanistic insight into stressor controllability effects. For instance, stress typically evokes an increase in cortisol and heart rate [49]. Physiological assessment could easily be incorporated in the experimental setup and would constitute further improvement to the paradigm. Fourth, as human research allows for the analysis of self-report data, we asked participants to supply various ratings. However, in the context of the triadic design, it is not a trivial task to phrase instructions in a way that allows for comparison of all three groups. Especially, the question of perceived control proved difficult. CC was not exposed to aversive stimulation which made the omission or rephrasing of some instructions necessary. We ensured comparability of perceived control ratings through equal phrasing of the item across groups (“How much control do you have over the task?”) at the cost of specifically asking EC and YC how much control they perceived over the termination of the stressor. Given that LH refers specifically to control over a stressor, our choice of phrasing may represent a serious drawback. However, true to the classic animal literature, we manipulated stressor controllability, not task controllability. Even if the assessment of perceived control was not ideal, we should still be able to infer that the observed group differences were due to our manipulation of stressor controllability. If comparing only EC and YC, future studies should explicitly assess perceived control over the stressor. Fifth, stressor controllability effects may vary, in respect to duration and intensity of stress induction as well as the length of time elapsed between exposure and subsequent assessment. In this regard, translating animal protocols proves near impossible. Stress research on humans is subject to thorough ethical scrutiny, as is research on animals. However, animal stress induction paradigms are often in the order of days or weeks [50], either because prolonged stress exposure is critical to the study subject (e.g., chronic antidepressant effects) [51,52] or because it is more ecologically valid. For instance, chronic stress is commonly discussed as a precursor to disorders such as depression and anxiety [53]. Hence, animal models that are constrained to acute stress exposure may not fully capture the underlying mechanisms [54]. Even if human research cannot easily employ a prospective design to study the long-term effects of experimentally induced controllable and uncontrollable stress, future studies could vary the time interval between stress exposure and outcome tests. It may be the case that control experience requires some processing before it exerts full effects on affective state, cognition, and behaviour. Note that our paradigm was not designed to induce a pathological state of learned helplessness which would clearly be unethical. Nonetheless, we were able to show that manipulation of stressor controllability, using a simple acoustic noise stressor, was effective in inducing significant effects in a very healthy sample. In a next step, it would be interesting to examine clinical populations and employ this paradigm to advance our knowledge of how control is implicated in mechanisms of both disease and resilience.

## 4. Materials and Methods

### 4.1. Study 1: Initial Paradigm

#### 4.1.1. Participants

A total of *N* = 87 healthy participants aged 18–40 took part. Recruitment included a semi-structured telephone interview similar to the SCID-I to ensure that participants had no current or lifetime DSM-IV axis I disorders. Because the study involved a reading task, we excluded individuals with dyslexia. The complex laboratory setup during parallel testing of multiple participants in the second testing session (Section 4.1.2) led us to constrain recruitment to right-handed individuals only. Further exclusion criteria comprised poor German language skills, current pregnancy, cardiac problems, tinnitus, and any other circumstances putting participants at risk during the stress induction procedure. Two participants broke off testing and three were excluded from analysis because of faulty experiment setup, poor German skills, and figuring out the experimental manipulation, respectively. Another two were excluded because health risks came up and they did not complete testing. All remaining participants were randomly assigned to three experimental groups: (1) EC (*n* = 27, age: *M* = 23.48, *SD* = 3.74, 56% female), (2) YC (*n* = 26, age: *M* = 25, *SD* = 4.26, 58% female), and (3) CC (*n* = 27, age: *M* = 25.85, *SD* = 4.3, 56% female). The study was approved by the ethics committee of the Institute of Psychology, Johannes Gutenberg University Mainz (2017-JGU-psychEK-003, 26/5/2017) and was conducted in accordance with the declaration of Helsinki. All participants provided written informed consent and were remunerated with 40 EUR.

#### 4.1.2. Procedure

Participants attended two testing sessions. In the first session, they were provided with information on the study and planned procedures were explained briefly. We then assessed demographic variables and other questionnaires (not reported here) via the online platform Social Science Survey [55]. In addition, working memory was measured using the German version of the Wechsler Adult Intelligence Scale [56]. Finally, participants completed a test of alertness which is part of the Tests of Attentional Performance battery [57]. The first session lasted 2 h. The second session was conducted in small groups of up to three participants—all assigned to the same experimental condition (EC/YC/CC). Participants were seated at their own desks, with walls restricting their field of view to their respective computer screens. We reminded participants that our research aimed at better understanding how individuals deal with stress. Therefore, they would be exposed to stressful stimulation in some of the tasks (CC was exposed to stress during the escape behaviour test, Section 4.1.4). Concerning stress induction (Section 4.1.3), EC and YC were told they could terminate the stressor, whereas CC participants were informed that they could effectively shorten trials to finish the task quicker. All participants were informed that their participation was voluntary and that they could stop the experiment at any time. First, participants reported their current mood, levels of anxiety, and depression (pre-stress assessment). Next, we calibrated shock intensity and participants underwent the stress induction procedure (Section 4.1.3). This was followed by a post stress assessment of affect, a test of escape behaviour, and a reading span task. Finally, YC participants were debriefed and informed that—contrary to instruction—their responses had had no effect on stress durations. They subsequently re-consented. The second session also lasted 2 h.

#### 4.1.3. Stress Induction

EC and YC were subjected to escapable or yoked inescapable aversive stimulation, respectively. The stressor consisted of electric shocks combined with intermittent 100ms white noise bursts at 85dB. Shocks were administered via a Digitimer DS7A current stimulator through an electrode attached to the back of the participant’s left hand. Acoustic noise was conveyed via Sennheiser HD 380 Pro headphones. At the beginning of data collection, we employed a decibel meter to verify our decibel threshold and to ensure participants’ safety. While the acoustic stressor was the same across participants, electric shocks were individually calibrated. After determining the threshold of perception, shock intensity was increased in 0.5 mA increments. Participants were able to administer shocks themselves by pressing a button. They rated each shock on a continuous paper-pencil scale with four anchors: “barely perceptible”, “unpleasant”, “very unpleasant”, and “really painful”. We encouraged participants to give honest responses by emphasising natural diversity in stimulus perception. Upon reaching a shock intensity that the participant rated as very unpleasant, but not painful (approx. 7–7.5 out of 10), the calibration process was stopped. Shock intensity was maintained at this established threshold throughout the session. One-to-one yoking of EC and YC participants ensured equal number of shocks and duration of white noise across both groups.

Participants underwent 40 trials of stress exposure and were instructed to terminate aversive stimulation by pressing the space bar. In each trial, they received four shocks during six seconds of intermittent noise before a cue prompted them to react. For EC, stressful stimulation was immediately stopped if a response was given within one second after cue onset. In case of missing or late responses, aversive stimulation continued up to a maximum of 15 s. Trials otherwise ended upon stressor termination and were followed by an inter-trial interval lasting one second ±250 ms jitter. For each YC participant, the stress duration was predetermined based on the responses of the yoked EC participant. Moreover, cue onset was shifted forward in time such that YC participants experienced continued aversive stimulation regardless of their reaction. We uniformly distributed cue onsets within four seconds to one second before the yoked EC participant’s reaction, which determined the end of stress exposure. This resulted in aversive stimulation stopping immediately or soon after button press in some trials, but continuing for a few seconds in others. Thus, YC participants perceived the termination of the stressor as arbitrary and outside of their control. Notwithstanding this group-specific manipulation, our yoking ensured equal stress duration across EC and YC. CC received no aversive stimulation, but shock intensity was nevertheless calibrated. For better comparability, CC participants performed the task with the electrode attached and wearing headphones.

**Figure 4:**
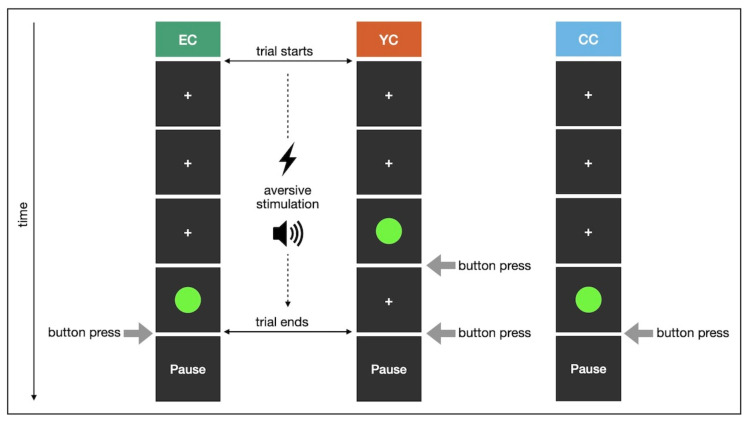
Stress induction in study 1 (one trial; EC participant successfully terminated aversive stimulation).

This experiment was implemented in Python 2.7 [58], using mainly functionalities of the PsychoPy package [59]. Figure 4 illustrates the stress induction procedure.

#### 4.1.4. Measurements

##### Ratings and RT during Stress Exposure

During the stress induction, we tracked participants’ RTs and, after every 10th trial, they supplied ratings to the following questions: (1) How aversive do you find the stressor? (2) How much control do you have over the task? (3) How exhausted do you feel? (4) How frustrated do you feel? (5) How helpless do you feel? We used a Likert scale ranging from 1 (not at all) to 7 (very). Note that we asked participants to rate perceived control over the task, not the stressor. This enabled us to compare ratings between all groups, including CC.

##### Pre-Post Affect Questionnaires

Approximately 10 min before and immediately after stress induction, participants completed two different questionnaires assessing state anxiety and depression, positive affect, and negative affect. The State-Trait Anxiety-Depression Inventory (STADI; [60]) consists of two sub scales for which excellent internal consistencies have been reported: anxiety (Cronbach’s *α* = 0.90) and depression (Cronbach’s *α* = 0.87) [61]. We used the STADI total score as an indicator of global state anxiety and depression. In addition, we examined total scores of the two subscales of the Positive and Negative Affect Schedule (PANAS; [62]) as indicators of positive (PANASpos; Cronbach’s *α* = 0.85) and negative affect (PANASneg; Cronbach’s *α* = 0.86), respectively.

##### Escape Behaviour Test

Highlighting our translational approach, we designed a new task inspired by the animal shuttle box paradigm [4,5,63]. This task assesses passivity and anxiety—behaviours commonly observed following uncontrollable stress in animal studies. After previous exposure to electric foot shocks, animals are placed in a two-compartment environment in which they, again, receive shocks. They can escape by shuttling from one compartment to the other, jumping a small barrier [5]. Specifically, the task requires some complexity (e.g., a two-way shuttle) in order to observe LH effects. Our escape behaviour test (Figure 3a) focused on translating important features of the shuttle box paradigm: free exploration of an open space without stress exposure, exposure to a familiar stressor, termination of this stressor through the participant’s action, and sufficient complexity of the escape task to observe LH effects. A 15 × 15 grid was displayed on-screen, which participants were instructed to navigate, using the arrow keys. Their location was marked by a lighter shading of the respective grid cell. Four “safe spaces” were hidden at random locations on the grid and lit up green if encountered. As subjects had to learn these four random safe locations, we were able to include the element of task complexity, necessary to observe LH effects. Participants were informed that exposure to a stressor was possible, but no further instructions were given. They completed a total of five trials, starting in the middle of the grid each time. Trials started with a ten-second stress-free phase in which participants could freely explore the grid. In a subsequent stress phase, EC and YC encountered the familiar aversive stimulation, whereas CC experienced this stressor for the first time. Stress exposure lasted either until the trial ended after a maximum of 20 s or until the participant moved onto a safe space. Safe space locations remained the same throughout the task. Based on animal research, we chose three measures as indicators of proactive escape behaviour: (a) exploration—number of unique spaces visited within the stress-free phase; (b) escape rate—number of escapes, i.e., successful termination of the stressor by finding a safe space; and (c) efficiency—number of escapes in relation to the amount of activity (sum of moves within time spent on the task). Higher scores indicate less efficient behaviour.

##### Working Memory

To assess working memory performance, we used a reading span task [64]. Reading span tasks are established measures of working memory capacity [65,66]. In short, participants were instructed to memorise and later recall single words which appeared in between unrelated sentences. For each sentence, participants indicated via button press whether or not it made sense. We employed partial-credit load scoring, which is frequently used in working memory research. The partial-credit load score represents the sum of correctly recalled elements, allowing for little spelling mistakes (for a detailed description, see [65]).

Both the escape behaviour task and the reading span task were implemented in Python 2.7 [58].

#### 4.1.5. Statistical Analysis

We performed a priori comparisons to verify that groups did not differ in sex, age, alertness, or working memory IQ. To assess differences in sex distribution, we computed a chi-square test. For all other comparisons, we employed one-way ANOVAs with group as the between-subject factor, or a Kruskal–Wallis test, if more appropriate. We then examined reported stressor aversiveness and perceived control as manipulation checks for our stress induction and manipulation of stressor controllability, respectively. To account for changes in ratings over trials, we conducted separate two-way repeated measures ANOVA (rmANOVA) with group (stressor aversiveness: EC, YC; perceived control: CC, EC, YC) as the between-subject factor and time (10th, 20th, 30th, 40th trial) as the within-subject factor. Using one-way ANOVA or a Kruskal–Wallis test, we separately investigated group differences in measurements assessed under acute stress (helplessness, exhaustion, frustration, and RT). To account for family-wise error due to the many dependent variables, we applied Bonferroni correction. Next, we investigated stress- and controllability-related effects on subsequently (post stress) measured dependent variables (changes in affective state, escape behaviour, and working memory). We analysed changes in affective state (global STADI score, PANASpos, PANASneg) from pre to post stress induction, using rmANOVAs with group as the between-subject factor and time (pre, post) as the within-subject factor. To investigate escape behaviour indices, we computed scores for exploration, escapes, and efficiency (Section 4.1.4). Since we focused on group differences in exploration during the stress-free phase and in escapes and efficiency during the stress phase, we employed one-way ANOVA or a Kruskal–Wallis test for these analyses. In a final step, we investigated partial-credit load scores as indicators of working memory, using one-way ANOVA. We also applied Bonferroni correction to account for multiple dependent variables in our investigation of stress- and controllability-related effects on subsequently (post stress) measured dependent variables. Only corrected significance levels are reported.

In general, we used box plots to check for outliers. We excluded only extreme outliers (values above third quartile plus three times the interquartile range (IQR) or below the first quartile minus three times IQR). Furthermore, we set our level of significance at *p* = 0.05. For rmANOVAs, we applied Greenhouse–Geisser correction in case of non-sphericity. Post hoc comparisons were assessed using paired *t*-tests, with Holm–Bonferroni correction [67] applied for multiple testing. Effect sizes are reported as partial *η*^2^ [68] for ANOVAs and *η*^2^*_H_* for Kruskal–Wallis tests [69]. Further analyses including time and sex effects are reported in the Supplementary Materials. Data were analysed in R (version 3.6.2; [70]) using the “rstatix” [71], “afex” [72], and “lsmeans” package [73] mainly.

### 4.2. Study 2: Improved Paradigm

#### 4.2.1. Participants

A total of 122 healthy participants aged 18–41, were recruited using the software ORSEE [74] and they took part in testing sessions in the Mainz Behavioral and Experimental Laboratory (MABELLA) at Johannes Gutenberg University Mainz. Due to time constraints, we decided to use this facility, which offers automated recruitment and allows for simultaneous testing of up to 25 participants. Exclusion criteria comprised current or lifetime mental disorder, poor German language skills, and hearing problems. In total, 106 participants provided complete data; six participants, however, were excluded because they reported current or lifetime mental disorders. The remaining sample consisted of *N* = 100 participants, who were randomly assigned to two experimental groups, an EC (*n* = 62, *M* = 22.77 years, *SD* = 3.99, 53% female) and a YC (*n* = 38, *M* = 24.26 years, *SD* = 4.2, 58% female). To allow for the analysis of inter-individual differences within EC, more participants were assigned to this group. However, we were unable to collect a sufficiently large sample (*N* > 80) within the duration of this project and such analyses also lie beyond the scope of this article. The unequal group sizes were considered in the statistical analysis (Section 4.2.5). Because the stressfulness of the aversive stimulation had been successfully established in study 1, we did not include a control group this time. Study 2 specifically aimed at improving the experimental manipulation of stressor controllability and was expected to result in distinct read-outs for EC and YC. EC was tested first and we used their average stress durations during the stress induction for pseudo-yoking (Section 4.2.3). The study was approved by the ethics committee of the Institute of Psychology, Johannes Gutenberg University Mainz (2017-JGU-psychEK-003, 26/5/2017) and it adhered to the declaration of Helsinki. All participants provided written informed consent and were remunerated with 9 EUR/hour.

#### 4.2.2. Procedure

Participants attended a 1.5 h group testing session, including up to 25 people. First, they filled in online questionnaires pertaining to demographic variables and current wellbeing. Following this, participants completed a pre-stress assessment of affect. The procedure was shorter but overall equivalent to study 1, i.e., participants then (1) underwent a stress induction, during which they rated perceived stressor aversiveness, controllability, exhaustion, and feelings of helplessness; (2) they reported their post stress affect; and (3) performed the escape behaviour test. Upon completion of the session, YC participants were debriefed and they re-consented.

#### 4.2.3. Stress Induction

The stress induction procedure differed from the setup described in Section 4.1.3 in a number of features. We were interested in seeing whether acoustic noise alone might serve to induce significant stress. In contrast to electric shocks, noise requires a less involved experimental setup and can be presented more readily. The acoustic noise used in study 1—verified by decibel meter—was delivered over AKG K81 DJ headphones.

Three different cues (circle, triangle, square) were included in the button-press task, each displayed equally often across 60 trials. Participants were instructed to match one of three buttons to each cue. They were told to figure out the correct button–cue combination which would stop aversive stimulation. Using a trial and error approach, they were bound to initially give some wrong responses and experience continued stress exposure. This served to enhance EC’s perception of action–outcome contingencies and enhance participants’ feeling of control. In EC, correct responses immediately terminated the stressor, whereas incorrect or slow responses led to continued aversive stimulation up to a maximum of ten seconds. However, in order to further increase perceived control and the experience of shortened stress durations in EC, we decreased the maximum stress duration by 500 ms after every 15th trial. In YC, participants’ responses had no effect on the stressor because stress durations were predetermined through pseudo-yoking. Specifically, the stress duration for each trial was set to the average obtained from EC. Additionally, we randomly shuffled mean durations to enhance the perception of arbitrary stress durations in YC. Pseudo-yoking is well-established in animal designs [50,75] and more practical in group testing sessions. All other parameters (response limit in EC, shift of cue onsets in YC) were the same as in study 1.

#### 4.2.4. Measurements

During stress induction, participants’ RT was measured. After every 15th trial, participants rated stressor aversiveness, perceived controllability, exhaustion, and feelings of helplessness (four ratings each). Immediately before and after stress induction, participants completed the PANAS and STADI questionnaires. After stress exposure, participants performed the escape behaviour test (Section 4.1.4). However, the task was improved by several modifications. Participants completed two blocks with five trials each. To condense the task, we shortened the free exploration phase to five seconds and the stress phase lasted up to five seconds or until a safe space was found. In addition, the participant’s position was reset to the middle space at the beginning of each new phase. Thus, we could directly compare behaviour during free exploration and stress exposure. Following the last trial of the first block, safe spaces were set to new random locations on the grid for the duration of the second block. Hence, participants were required to search anew for the safe places. This modification served to introduce a lack of control element. We sought to examine whether previous stressor controllability experience would lead to divergent reactions. We did not assess participants’ working memory in this study.

#### 4.2.5. Statistical Analysis

We performed a priori comparisons to verify that groups did not differ in sex, age, or level of education using a chi-square test, a *t*-test for independent samples, and the Wilcoxon rank sum test, respectively. As in study 1, we then examined stressor aversiveness and controllability ratings to ascertain successful stress induction and manipulation of stressor controllability. For analysis of group differences during acute stress exposure (helplessness, exhaustion, and RT), we used *t*-tests for independent samples to compare means. As in study 1, we applied Bonferroni correction to account for family-wise error. Next, we conducted separate rmANOVAs with group as the between-subject factor and time (pre, post) as the within-subject factor to analyse changes in affective state (global STADI score, PANASpos, PANASneg) from pre to post stress induction by group. Finally, as in study 1, we calculated exploration, escapes, and efficiency scores in the escape behaviour test. Exploration during the stress-free phase was analysed using rmANOVA with group as the between-subject factor and block (1, 2) as the within-subject factor. We computed escapes and efficiency across trials and examined only data pertaining to the stress phase, thus, employing rmANOVAs with group as the between-subject factor and block as the within-subject factor. Again, we corrected for family-wise error in our analysis of transfer stress effects during subsequent assessments, using Bonferroni correction.

As in study 1, we excluded extreme outliers and set our level of significance at *p* = 0.05. For rmANOVAs, we applied Greenhouse–Geisser correction. If residuals markedly deviated from normality, we employed a robust rmANOVA using the WRS2 package [76] and reported the effect size measure suggested by Algina and colleagues [77]. In general, we accounted for unequal group sizes by confirming the results using robust rmANOVA. Post hoc comparisons were assessed using paired *t*-tests, with Holm–Bonferroni correction [67] applied for multiple testing. Effect sizes are reported as partial *η*^2^ [68] for ANOVAs, *η*^2^*_H_* for Kruskal–Wallis test [69], and Cohen’s *d* [78] for *t*-tests. Further analyses including time and sex effects are reported in the Supplementary Materials. As in study 1, analyses were conducted in R (version 3.6.2; [70]).

## 5. Conclusions

Translational research is essential to assess the extent to which animal findings replicate in humans. We developed and validated a new stress paradigm that translates the animal LH protocol to human research. Our final manipulation robustly induced differential effects in behaviour, but we did not observe effects on changes in affective state, and putative differences in cognitive performance remain to be tested. An independent study incorporating affective state measurements as well as cognitive and behavioural tasks would certainly be useful. Our paradigm shows promise as a feasible and flexible way to study the different effects of controllable and uncontrollable stress on various levels. Future studies could employ a further improved version of our design to promote clarification of the mechanisms by which control experience moderates our reaction to and appraisal of stressful events. Such research serves to improve our understanding of the aetiology of mental disorders and opens up new perspectives on resilience mechanisms. Applications might also inspire back-translation, thus, fostering fruitful and necessary synergies between human and animal research.

## Figures and Tables

**Table 1:** A priori group comparisons in study 1.

Variable	Group			Statistical Test	*p*	Effect Size
	EC	YC	CC			
*n* ^1^	27	26	27			
Sex (% Female)	56	58	56	Chi-Squared	0.984	*X*^2^ = 0.03
Mean Age (*SD*)	23.48 (3.74)	25 (4.26)	25.85 (4.3)	Kruskal–Wallis	<0.05	*η*^2^*_H_* = 0.06
Mean Working Memory IQ (*SD*)	100.89 (9.55)	104.58 (11.53)	104.3 (13.99)	One-Way ANOVA	0.45	Partial *η*^2^ = 0.02
Mean Alertness (*SD*)	45.3 (6.31)	43.96 (6.99)	42.11 (6.31)	One-Way ANOVA	0.24	Partial *η*^2^ = 0.04

^1^ Note. *N* = 80.

**Table 2:** A priori group comparisons in study 2.

Variable	Group		Statistical Test	*p*	Effect Size
	EC	YC			
*n* ^1^	62	38			
Sex (% Female)	53	58	Chi-Squared	0.80	*X*^2^ = 0.06
Mean Age (*SD*)	22.77 (3.99)	24.26 (4.2)	*t*-test	0.08	*d* = −0.36
Mean Level of Education (*SD*)	7.84 (1.43)	8.03 (1.55)	Wilcoxon Rank Sum	0.51	*r* = 0.07

^1^ Note. *n* = 100.

## Supplementary Materials

Supplementary materials can be found at https://www.mdpi.com/1422-0067/21/17/6010/s1.

## Abbreviations

LH	Learned helplessness
EC	Escapable stress condition
YC	Yoked inescapable stress condition
CC	Control condition
