# Supplementary material for: A Translational Paradigm to Study the Effects of Uncontrollable Stress in Humans
Source: Int J Mol Sci. 2020 Aug 20;21(17):6010. doi: 10.3390/ijms21176010 (PMC7503322; doi:10.3390/ijms21176010)
Supplement: Supplementary file 1 [file ijms-21-06010-s001.pdf]

Supplement

A Translational Paradigm to Study the Effects of Uncontrollable Stress in Humans

Laura E. Meine [1], Katja Schueler [1], Gal Richter-Levin [2,3,4], Vanessa Scholz [1,5] and Michèle Wessa [1,6,*]

[1] Department of Clinical Psychology and Neuropsychology, Institute of Psychology, Johannes Gutenberg-University Mainz, 55122 Mainz, Germany
[2] Department of Psychology, University of Haifa, 3498838 Haifa, Israel
[3] Sagol Department of Neurobiology, University of Haifa, 3498838 Haifa, Israel
[4] The Integrated Brain and Behavior Research Center (IBBR), University of Haifa, Mount Carmel, 3498838 Haifa, Israel
[5] Donders Institute for Brain, Cognition and Behaviour, Radboud University, 6525 HR Nijmegen, Netherlands
[6] Leibniz Institute for Resilience Research, Research Group Wessa, 55122 Mainz, Germany

* Correspondence: wessa@uni-mainz.de

This supplementary material has been provided by the authors to give readers additional information about their work. Data and analysis code are available on the Open Science Framework: https://osf.io/pw4m5/.

Supplement 1
Frustration ratings under acute stress exposure in study 1

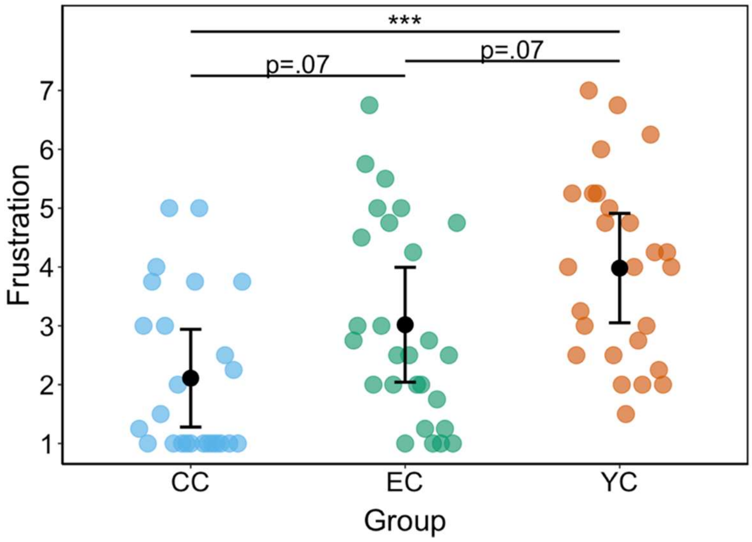

Figure S1. Frustration ratings during acute stress exposure by group. Error bars denote standard error.

Supplement 2

Exploration in the escape behaviour test by group in study 1

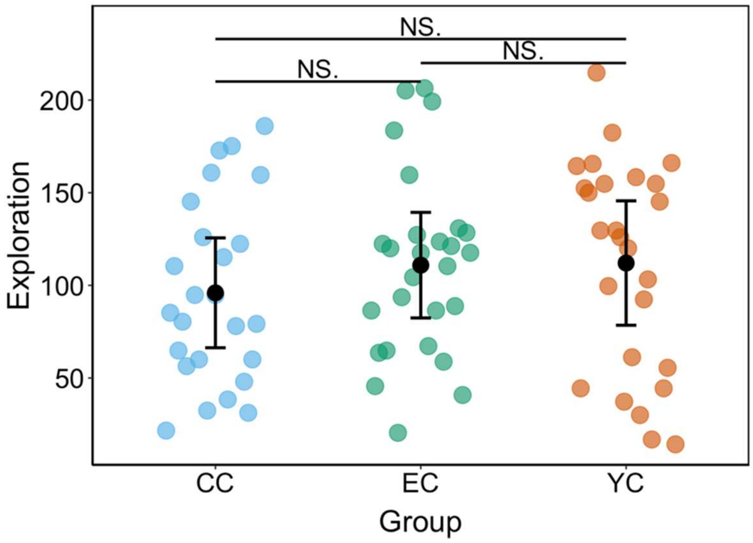

Figure S2. Exploration during the stress-free phase in the escape behaviour test by group. Error bars denote standard error.

Supplement 3

Escapes from stress in the escape behaviour test by group in study 1

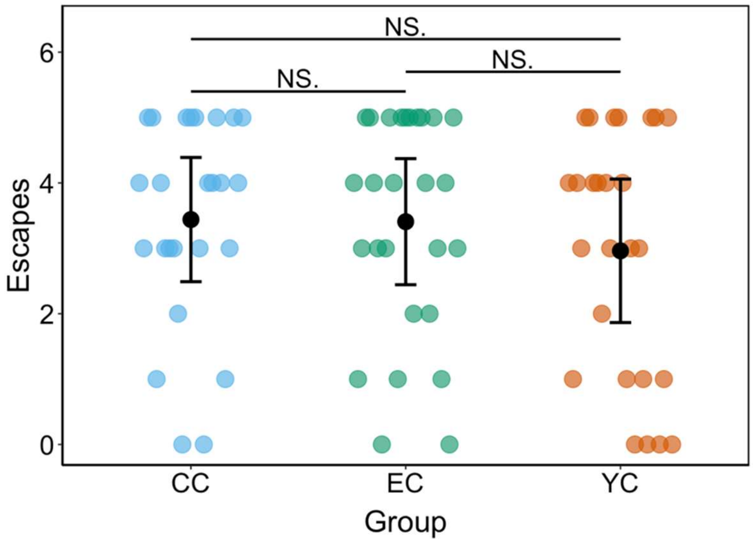

Figure S3. Escapes from stress in the escape behaviour test by group. Error bars denote standard error.

Supplement 4

Efficiency under stress in the escape behaviour test by group in study 1

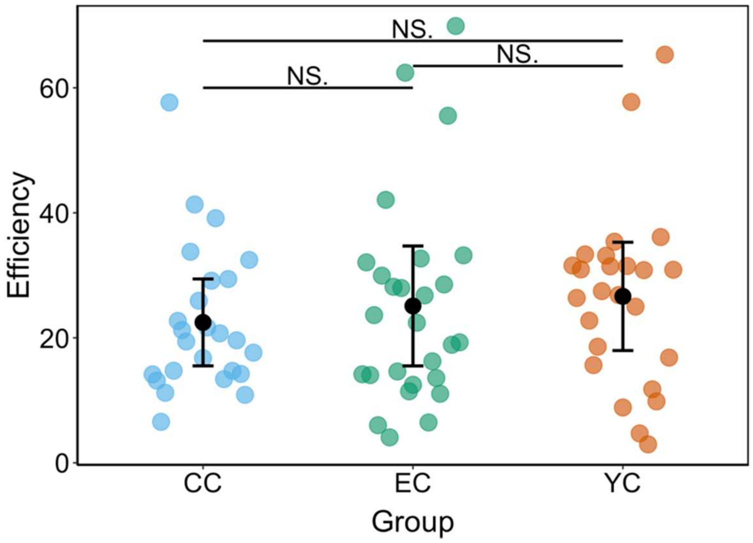

Figure S4. Efficiency under stress in the escape behaviour test by group. Error bars denote standard error.

Supplement 5

Working memory by group in study 1

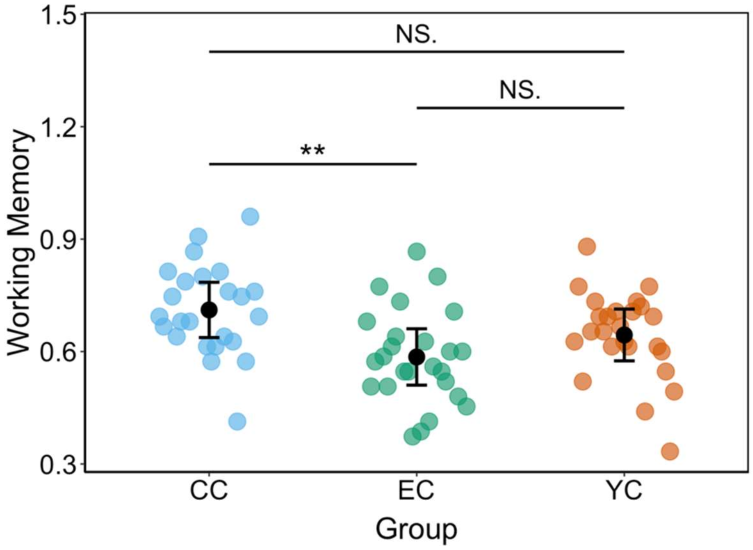

Figure S5. Working memory following the stress induction by group. Error bars denote standard error.

Considering that the paradigms described in study 1 and study 2 represent the development of a new design, we report exploratory analyses of time and sex effects on ratings and reaction times under acute stress. We conducted separate repeated measures ANOVA with group (study 1: EC, YC, CC; study 2: EC, YC) and sex (male, female) as between-subject factor and time (ratings: 10th, 20th, 30th, 40th trial; RT: 1-40 trials) as within-subject factor. Furthermore, we explored sex differences in subsequent assessments, i.e. in respect of changes in affective state, escape behaviour, and working memory. Results are reported in the following paragraphs. We hope that these additional analyses will be useful to future applications.

Supplement 6

Effects of sex on stressor aversiveness ratings and perceived control in study 1

Men reported lower stressor aversiveness compared to women ($F(1, 45) = 14.70$, $p < .001$, partial $\eta^2 = 0.25$). Concerning perceived control, no significant sex differences emerged ($F(1, 72) = 2.71$, $p = .10$, partial $\eta^2 = 0.04$). However, we observed a significant three-way-interaction between group x sex x time on perceived control ($F(6, 213) = 4.27$, $p < .01$, partial $\eta^2 = 0.11$ (Greenhouse-Geisser corrected, $\varepsilon = 0.65$), s. Figure S6 below).

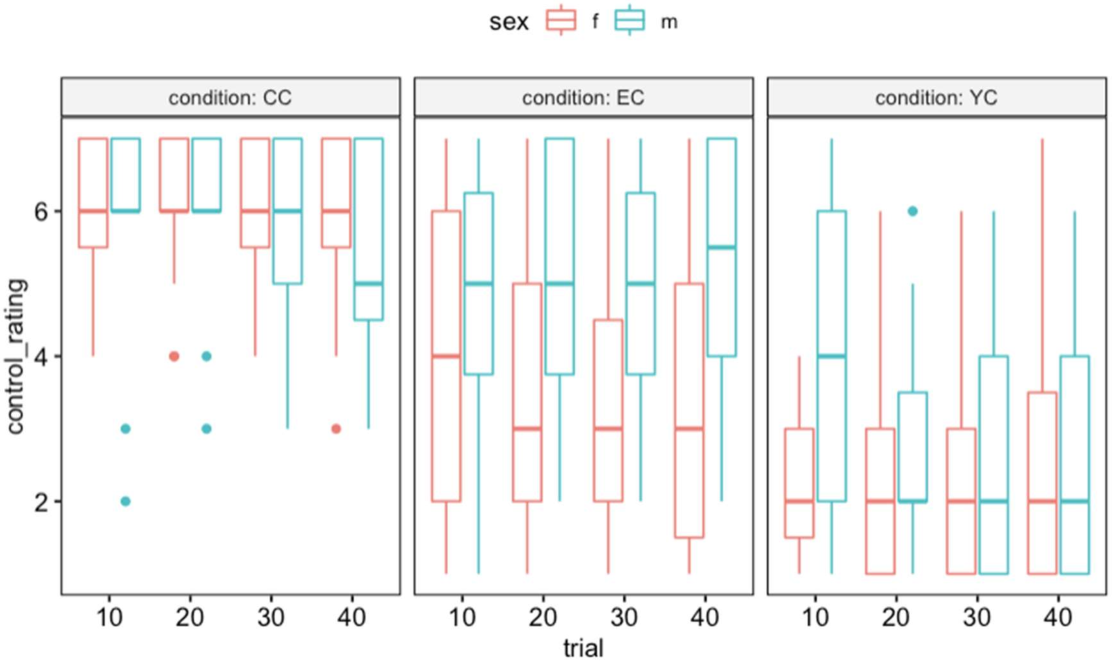

Figure S6. Boxplots split by group (CC, EC, YC) and sex across trials showing perceived control ratings.

Supplement 7

Effects of time and sex under acute stress exposure in study 1

Helplessness ratings remained constant over time ($F(3, 216) = 0.45$, $p < .62$, partial $\eta^2 = 0.006$ (Greenhouse-Geisser corrected, $\varepsilon = 0.59$)). Exhaustion ratings slightly increased ($F(3, 216) = 4.29$, $p < .05$, partial $\eta^2 = 0.06$ (Greenhouse-Geisser corrected, $\varepsilon = 0.63$)). Concerning frustration, we observed a significant interaction of group x time ($F(6, 216) = 3.00$, $p < .05$, partial $\eta^2 = 0.08$ (Greenhouse-Geisser corrected, $\varepsilon = 0.80$)). This finding reflected increased frustration over time in YC, whereas ratings stayed relatively constant in CC and EC. In respect of reaction times (RT), there was an effect of time ($F(39, 2652) = 3.49$, $p < .001$, partial $\eta^2 = 0.05$ (Greenhouse-Geisser corrected, $\varepsilon = 0.40$)), RT decreased sharply in the first few trials.

Analysis of helplessness ratings revealed a group x sex interaction ($F(2, 72) = 5.82$, $p < .01$, partial $\eta^2 = 0.14$). Whereas men and women did not differ in reported helplessness in CC, men rated less helplessness in both EC and YC, with ratings markedly differing between sexes especially in EC. We also observed a significant interaction effect of group x sex on exhaustion ratings ($F(2, 72) = 5.77$, $p < .01$, partial $\eta^2 = 0.14$). Men in both EC and YC reported less exhaustion compared to women, whereas, in CC, women reported less exhaustion. Across groups, men reported less frustration compared to women ($F(1, 72) = 9.35$, $p < .01$, partial $\eta^2 = 0.12$). No sex effects were found for RT ($F(1, 68) = 0.87$, $p = .35$, partial $\eta^2 = 0.01$).

Supplement 8

Effects of sex on subsequent assessment in study 1

Overall, men reported lower combined depressive state and anxiety compared to women ($F(1, 65) = 10.11$, $p < .01$, partial $\eta^2 = 0.14$). Concerning negative mood, men also displayed lower scores compared to women ($F(1, 65) = 14.32$, $p < .001$, partial $\eta^2 = 0.18$). With regard to positive mood, a trend for sex differences emerged ($F(1, 72) = 3.27$, $p = .07$, partial $\eta^2 = 0.04$). No sex differences were evident for indices of escape behaviour or working memory.

Supplement 9

Effects of sex on stressor aversiveness ratings and perceived control in study 2

We observed a three-way-interaction between group x sex x time on stressor aversiveness ratings ($F(3, 279) = 3.80$, $p < .05$, partial $\eta^2 = 0.04$ (Greenhouse-Geisser corrected, $\varepsilon = 0.77$); s. Figure S7 below). No sex differences emerged for perceived control ratings ($F(1, 94) = 1.55$, $p = .22$, partial $\eta^2 = 0.02$).

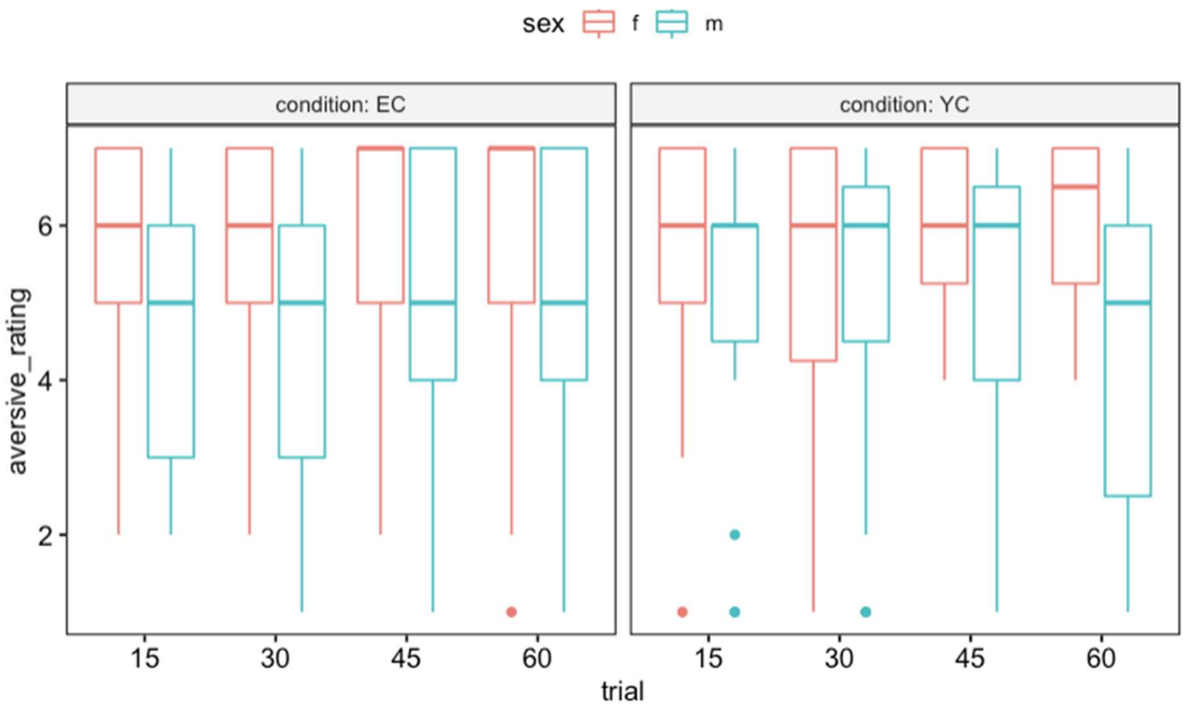

Figure S7. Boxplots split by group (EC, YC) and sex across trials showing stressor aversiveness ratings.

Supplement 10

Effects of time and sex under acute stress exposure in study 2

Helplessness ratings decreased over time in both groups ($F(3, 282) = 9.20$, $p < .001$, partial $\eta^2 = 0.09$ (Greenhouse–Geisser corrected, $\varepsilon = 0.88$)). There was a significant effect of time on exhaustion ($F(3, 282) = 6.95$, $p < .001$, partial $\eta^2 = 0.07$ (Greenhouse–Geisser corrected, $\varepsilon = 0.77$)). Across groups, reported exhaustion increased, then decreased again (s. Figure S8 below). There was no significant effect of trial on reaction times ($F = 0.96$, $p = .57$, $\delta_R = -0.25$).

No effects of sex were observed on helplessness ratings ($F(1, 94) = 0.90$, $p = .35$, partial $\eta^2 = 0.01$) but men reported less exhaustion compared to women ($F(1, 94) = 9.43$, $p < .01$, partial $\eta^2 = 0.09$). Reaction times did not differ between men and women.

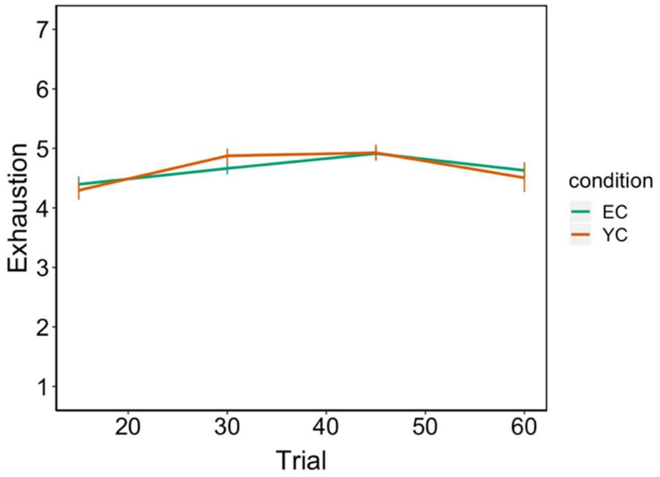

Figure S8. Exhaustion ratings over trials split by group (EC, YC).

Supplement 11

Effects of sex on subsequent assessment in study 2

Concerning depressive state and anxiety, no sex differences emerged ($F(1, 94) = 0.13$, $p = .71$, partial $\eta^2 = 0.001$). We observed a three-way-interaction between group x sex x time on negative mood ratings ($F(1, 93) = 8.74$, $p < .01$, partial $\eta^2 = 0.09$; s. Figure S9 below). There was a sex x time interaction on positive mood ($F(1, 94) = 5.28$, $p < .05$, partial $\eta^2 = 0.05$), reflecting less decrease in positive mood in men compared to women.

With regard to exploration in the escape behaviour test, we observed a significant sex x block interaction ($F(1, 94) = 4.99$, $p < .05$, partial $\eta^2 = 0.05$). Men showed a greater increase in exploration from block 1 to block 2 compared to women. Even so, men did not escape the stressor more often than women — there was no significant difference in escape rate ($F(1, 94) = 2.50$, $p = .12$, partial $\eta^2 = 0.03$). Analysis of efficiency revealed a significant sex x block interaction ($F(1, 89) = 5.73$, $p < .05$, partial $\eta^2 = 0.06$). Whereas, men became slightly more efficient over blocks, women behaved less efficiently in block 2 compared to block 1.

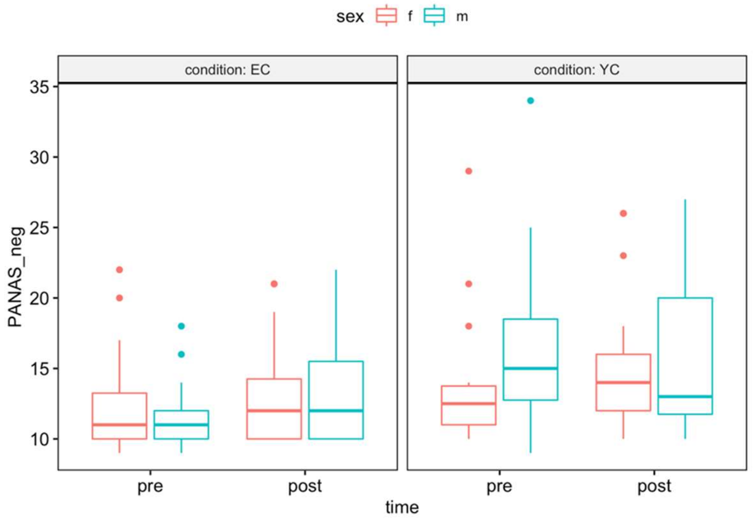

Figure S9. Boxplots split by group (EC, YC) and sex across mood assessments (pre and post stress exposure) showing differential changes in negative mood.
